# Profiling Patients with Chronic Ulcers Using K-Means Clustering and Analysis of the Impact on the Consumption of Medical Resources: Retrospective Study on Hospitalized Patients in Romania

**DOI:** 10.3390/jcm14176252

**Published:** 2025-09-04

**Authors:** Mona Taroi (Yassin Cataniciu), Ilie Gligorea, Radu Fleacă, Liliana Vecerzan (Novac), Andrada Prihoi, Carmen-Daniela Domnariu

**Affiliations:** 1 Faculty of Medicine, Lucian Blaga University of Sibiu, 550169 Sibiu, Romania; 2 Department of Technical Sciences, Faculty of Military Management, Nicolae Bălcescu Land Forces Academy, 550170 Sibiu, Romania

**Keywords:** chronic wounds burden, patient profiling, early identification, healthcare resource utilization, unsupervised clustering

## Abstract

**Background/Objectives:** Chronic ulcers represent a major public health concern, being associated with substantial morbidity, impaired quality of life, and significant costs to healthcare systems. Against the backdrop of an aging population and increasing prevalence of chronic comorbid conditions, this study aimed to profile hospitalized patients with chronic ulcers in Romania and to examine their differential patterns of healthcare resource utilization. **Methods:** We conducted a retrospective analysis of the national administrative hospitalization database between 2017 and 2022, including adult patients with at least two admissions coded with a primary diagnosis of chronic ulcer. Sociodemographic, clinical, and healthcare utilization indicators were extracted, standardized, and analyzed using the K-means clustering algorithm to derive utilization-based phenotypes. **Results:** Two distinct patient clusters were identified: the first comprised predominantly elderly patients with multiple comorbidities, prolonged hospitalizations, and frequent readmissions, representing a high-burden profile; the second included relatively younger patients with fewer comorbidities, shorter hospital stays, and lower readmission rates, reflecting a more stable clinical profile. The high-burden cluster accounted for a disproportionate share of inpatient resource consumption, underscoring its impact on the healthcare system. **Conclusions:** These findings highlight the importance of early identification of potential high-burden patients, enabling the implementation of personalized care strategies and more efficient allocation of hospital resources, with the potential to improve health outcomes and support healthcare system sustainability.

## 1. Introduction

Chronic ulcers are defined as skin lesions that fail to progress through an orderly and temporal sequence of healing or that do not respond to standard therapies within three months [1]. These conditions represent a major challenge for health systems globally, generating substantial costs through the need for complex and long-term medical care, repeated hospitalizations and a significant negative impact on patients’ quality of life [2,3]. Their impact translates not only into direct consumption of resources, but also into indirect economic losses, determined by incapacity for work and the associated social burden. From an epidemiological point of view, there is an increase in the prevalence of chronic ulcers, a phenomenon closely related to the aging of the population and the increased incidence of predisposing pathologies, such as obesity, diabetes, peripheral arterial diseases and chronic venous insufficiency [4,5].

In this context, profiling patients with chronic ulcers becomes a pressing necessity. Detailed knowledge of the clinical-demographic characteristics of different subgroups of patients is fundamental for the development of personalized care strategies, adapted to the specific needs of each case [6,7]. Such an approach allows not only the optimization of therapeutic outcomes, but also a more efficient allocation of medical resources, often limited, directing them to high-risk patients or to the most cost-effective interventions.

The objective of this study was to derive an unsupervised, utilization-based taxonomy (phenotypes) of adult patients hospitalized with chronic ulcers in Romania, using sociodemographic characteristics and indirect indicators of disease severity, such as length of stay, readmission frequency, and comorbidity count. These burden-based profiles were then analyzed in relation to resource consumption, with the aim of estimating the associated economic burden and identifying high-risk groups. This approach is intended to support early risk stratification and inform more efficient, targeted public health and clinical management strategies.

## 2. Materials and Methods

### 2.1. Study Settings

This study was conducted using data from the Romanian national hospital administrative database, which covers all inpatient episodes in public hospitals. The Romanian healthcare system is a social health insurance system that provides universal coverage. Hospital care for chronic conditions like lower limb ulcers is delivered pre-dominantly by public hospitals and is reimbursed through a national Diagnosis-Related Group (DRG) [8] payment system. The dataset used in our analysis reflects resource consumption within this inpatient setting but does not capture care provided in primary or outpatient settings, which are funded and recorded separately. This context is important for interpreting our utilization-based phenotypes, which are defined by patterns of hospital resource consumption within this specific healthcare framework.

### 2.2. Study Population and Data Sources

The population analyzed in this study was composed of hospitalized patients with diagnoses of chronic ulcers, collected in a representative time frame for assessing the impact and severity of these conditions in hospital practice. The available data allowed the detailed characterization of the demographic, clinical and social aspects of patients, providing a solid basis for identifying differentiated risk profiles.

The data were collected from the administrative database of the National Institute of Public Health, which includes the reports of all state hospitals in Romania. The data set in this study consists of hospitalizations of adult patients (≥18 years), registered nationwide, between January 1st and 31 of December 2022, diagnosed with chronic ulcers.

ICD-10 codes [9] were used to identify chronic ulcers as the primary diagnosis at discharge. These diagnoses have been categorized into six principal disease groups: venous ulcer (I83.x—varicose veins of the lower extremities with ulceration), arterial ulcer (I70.23—atherosclerosis of the arteries of the extremities with ulceration), diabetic ulcer (E1x.73—diabetes mellitus with foot ulceration due to multiple causes), pressure ulcer (L89—pressure ulcer), unclassified foot ulcer (L97—ulceration of the lower limb not elsewhere classified), unclassified skin ulcer (L98.4—chronic ulceration of the skin not elsewhere classified). This classification allows both the specific analysis by major etiologies and a global assessment of the impact on the health system.

According to the standardized methodology in the international literature [10], patients were grouped by age ranges as follows: <45 years, 45–54 years, 55–64 years, 65–74 years, 75–84 years, ≥85 years. This division allows direct comparability with other epidemiological studies and facilitates the analysis of the distribution of burden by risk groups and life stages.

To ensure the study captured patients with genuinely chronic and clinically complex wounds, we included only individuals who had at least two hospitalizations over the six-year study period with a primary diagnosis of chronic skin ulcer. This criterion intended to reduce the risk of miscoding, misclassification, and potential DRG-driven upcoding of isolated acute wounds. By focusing on recurrently hospitalized patients, we targeted a subpopulation characterized by prolonged healing trajectories, frequent complications, and higher demand for multidisciplinary inpatient care [11].

For each patient, the following variables were extracted: demographic data: age at the time of admission, gender, place of residence (urban/rural), socio-economic status (employed, retired, unemployed, etc.); clinical data: type of ulcer and associated comorbidities, according to ICD-10 coding of primary and secondary diagnoses, healthcare resource utilization: duration of hospitalization (total number of days hospitalization per patient), number of hospitalizations. It is important to note that the administrative nature of the dataset limits access to certain clinical variables. Specific wound-related details such as ulcer size, anatomical location, duration prior to admission, or treatment modality were not available, so we focused on burden-based profiles constructed from sociodemographic characteristics and indirect proxies of clinical severity, including number of comorbidities, hospitalization duration and readmission count.

All analyses were conducted on a fully deidentified dataset. Patient level identifiers were removed prior to analysis, and only aggregated results are reported. Access to the data was granted under a data sharing agreement with the National Institute of Public Health, in full compliance with the European Union General Data Protection Regulation (GDPR) and national legislation. No individual level data can be shared publicly due to legal restrictions.

The nationwide administrative dataset was provided as a harmonized extract using standard ICD-10 coding and core administrative fields; hospital/center identifiers were removed by the data custodian as part of deidentification, so center level analyses are not possible with the present data.

### 2.3. Data Preprocessing

To achieve robust profiling of patients with chronic ulcers, the selection of variables included in the clustering analysis is an essential step, based on both clinical relevance and evidence from the literature [12,13,14]. The purpose of this selection is to identify those characteristics that significantly differentiate subgroups of patients and that can influence both the individual prognosis and the consumption of resources within the health system.

Demographic variables: age and sex are fundamental factors in the epidemiology and evolution of chronic ulcers. Age, entered as a continuous variable or in groups, allows the identification of clusters with specific risks associated with each life stage. Gender also influences epidemiological indices and the evolution of chronic ulcers, thus being indispensable for relevant segmentation. Additional variables such as the environment of residence (urban/rural) and socio-economic status, relevant for the assessment of epidemiological and evolutionary characteristics, were added.

Type of ulcer: identification and coding of ulcer types according to ICD-10 then grouping by categories: venous, arterial, diabetic, bedside scale, unclassified ulcer of the lower limb, chronic unclassified skin ulcer, allows precise segmentation of the studied population according to the predominant etiology, facilitating the differentiated analysis of clinical characteristics and resources used for each category.

Comorbidities: the presence of associated diseases, especially obesity, cardiovascular diseases and infections, plays a crucial role in the etiopathogenesis, evolution and prognosis of chronic ulcers. For each patient, the main comorbidities were extracted based on the secondary diagnoses associated with each discharge, thus allowing the assessment of their cumulative impact on the complexity of the case and on the consumption of resources.

Indicators of resource consumption: the total length of hospitalization (expressed as the total number of days spent in hospital during the study period) reflects the degree of complexity and severity of cases, being a proxy for the intensity of medical care required. The total number of admissions during the six years of the study, per patient, provides additional information on the prolonged evolution of severe cases with complications and recurrences.

The selection of these variables was made in accordance with the recommendations of the literature on profiling studies of patients with chronic diseases and is supported by evidence on the impact of each characteristic on clinical evolution and on the associated costs [15,16,17]. The careful choice of these variables ensures not only the clinical relevance of the identified clusters, but also the practical utility for resource allocation policies and for the development of personalized strategies for the management of patients with chronic ulcers.

Predictors and data elements: Clustering predictors included demographics (age, sex, residential background), comorbidity burden, and healthcare utilization (length of stay and number of admissions per patient). Although a variable indicating admission through the emergency room (ER) was available, we deliberately excluded it to avoid potential circularity between care process variables and outcomes. Our aim was to derive burden-based phenotypes using baseline characteristics and utilization patterns, rather than variables that may be downstream consequences of disease severity or care decisions.

The extract did not include referral origin (e.g., primary care or outpatient specialist), procedure/operating-theatre codes, or structured intensive care unit (ICU)/intermediate-care flags. ICU admissions were only partially identifiable through admission to intensive care specialties, which occurred infrequently and inconsistently. Admission type (emergency vs. elective) was available only as a text field and not used due to similar concerns over interpretability and redundancy with the utilization indicators already included.

The selection of predictors was guided by simplicity and practical relevance, based on four key principles:

- availability early in the admission process to avoid introducing bias from downstream care decisions;
- consistent and standardized capture within the national administrative database;
- interpretability and non-redundancy, ensured by examining correlations to avoid overlapping variables; and
- focus on indicators that reflect a patient’s overall burden on the hospital system.
- Starting from the nationwide administrative extract, we:
- defined the cohort using ICD-10 codes for chronic ulcers and excluded ineligible records;
- calculated length of stay and verified date consistency;
- removed duplicate patient and encounter IDs;
- aggregated admission-level data to the patient level (total LOS, number of admissions, comorbidity burden);
- applied z-score standardization to continuous predictors; and
- one-hot encoded categorical variables (sex and residential background).

This ensured that all features were numeric and comparable in scale, as required by the K-means algorithm. No imputation was necessary, as the dataset had already undergone institutional validation.

A critical step in the analysis of medical data is the evaluation and treatment of missing values, given the potential impact on the validity and robustness of statistical conclusions. In the present study, it was not necessary to implement techniques for dealing with missing values, as the dataset analyzed was subjected to a rigorous validation and verification process at institutional level. More specifically, the data comes from a standardized national administrative base, in which each admission record is complete, and the essential variables for the analysis were mandatory for hospital reporting. As a result, there were no missing values for the variables included in the profiling analysis, this peculiarity providing a solid basis for the application of clustering methods, without the risk of introducing bias through additional imputations or exclusions.

Subsequently, the additional validation carried out at the study level involved the inclusion of only patients with at least two hospitalizations for chronic ulcers, which ensures the consistency and integrity of the selected records.

To allow quantitative analysis and the application of clustering methods (K-means), the selected variables were processed and coded according to the following principles:

- Numerical variables: variables such as age (years), total length of hospitalization (days), total number of admissions. For some descriptive or cluster validation analyses, age was divided into standardized groups, and the results were interpreted on both raw and category data.
- Categorical variables: Sex was binary coded (0 = female, 1 = male), facilitating quick interpretation in statistical models. The category of ulcer was coded based on the six previously defined groups, with each patient associated with a label (e.g., 1 = venous ulcer, 2 = arterial ulcer, 3 = diabetic ulcer, 4 = pressure scale, 5 = lower limb ulcer not elsewhere classified, 6 = chronic skin ulcer not elsewhere classified).
- Additional variables: in the structure of the dataset there were additional ordinal or nominal variables (e.g., urban/rural residence environment), they were coded accordingly: nominal variables by one-hot coding or numerical labels, and ordinal variables according to natural order.
- Normalization of variables: to ensure comparability between variables with different scales and to improve the performance of the K-means algorithm, numerical variables were standardized with z-score [18] or normalized to the range [0,1], so that no variable dominates the clustering process by numerical size alone.

Through this systematic approach of coding and preprocessing, each case benefited from detailed framing from a demographic, etiological, and comorbidity point of view, allowing the obtaining of clinically relevant, easily interpretable and reproducible profiles. This method ensures the robustness of clustering analysis and interpretation of differences between resource consumption profiles, facilitates the extrapolation of results in similar clinical contexts and supports the substantiation of recommendations for the integrated management of chronic ulcers.

### 2.4. Clustering Methodology

#### 2.4.1. The Choice of the K-Means Method: Justification, Advantages and Limitations

In this research, we opted for the application of the K-Means unsupervised learning algorithm to identify distinct profiles of hospitalized patients with chronic ulcers. The choice of method is based on both the characteristics of the available data and the operational advantages of the algorithm in medical contexts, where population segmentation based on demographic, clinical and resource consumption traits can guide personalized decisions and the efficient allocation of health resources.

K-Means is a partition algorithm that divides observations into unordered k clusters, minimizing internal variation (intra-cluster) and maximizing separation between groups (inter-cluster). This method is well adapted for situations in which it is desired to highlight latent patterns in the numerical data, such as age, length of hospitalization, number of comorbidities, frequency of hospitalizations, etc.

Advantages of the K-Means method:

- High computational efficiency: The algorithm is scalable for large datasets with linear complexity relative to the number of observations.
- Intuitiveness and interpretability: the results (cluster centers) can be interpreted directly as prototypes of patient profiles.
- Flexibility: allows the integration of a variable number of clinically and logistically relevant numerical variables (e.g., age, days hospitalized, comorbidities).
- Popularity and robust implementation: It is available in a variety of validated libraries (e.g., scikit-learn) [19], with options for smart initialization (K-means++), which minimizes the risk of convergence to suboptimal local solutions.

Limitations of the K-Means method:

- The need to specify the number of k clusters: its choice involves auxiliary methods (e.g., the “elbow” method [20]), which can introduce subjectivity.
- Scaling sensitivity: The performance and shape of clusters can be influenced by unscaled variables or the presence of outliers.
- Implicit expectation of spherical and balanced clusters: in the medical context, distributions can be unbalanced (e.g., patients with multiple comorbidities vs. some with a simple profile), which can limit the fidelity of the segmentation.
- Inability to work efficiently with categorical variables requires conversions (e.g., one-hot encoding), which can complicate interpretability or lead to sparsity.

The choice of the K-Means method is therefore justified by an optimal compromise between analytical accuracy, computational complexity and clinical interpretability. Considering the main objective of the study, the identification of homogeneous groups of patients from the perspective of clinical risks and resource consumption, this method allows the exploration of latent models of population organization that can underpin stratification and targeted intervention policies in medical practice.

The steps mentioned in the data processing section led to the formation of a numerical, scaled and coherent dataset suitable for the application of unsupervised learning methods. This set allows the identification of homogeneous groups of patients, relevant from a clinical and operational perspective.

The optimal number of clusters (k) was determined using a rigorous combination of quantitative validation metrics and a focus on clinical interpretability. We computed three distinct metrics [21], for k values ranging from 2 to 5: the Silhouette score, the Davies-Bouldin index, and the Calinski-Harabasz index. The results of this analysis are presented in detail in Table 1.

**Figure 1:**
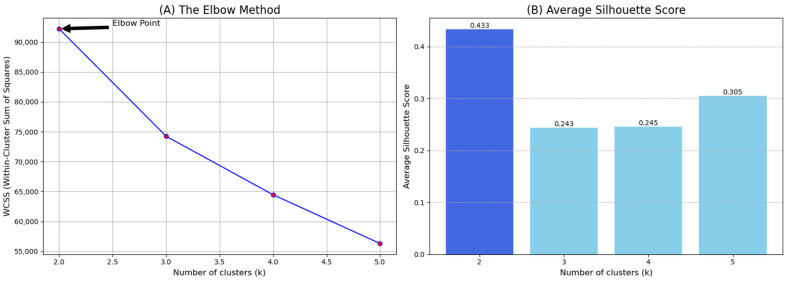
Elbow method inflection point at k = 2; Silhouette score was highest for k = 2 (0.433).

As shown in Figure 1, the Elbow method revealed a clear inflection point at k = 2, indicating diminishing returns in variance explanation for additional clusters. This was strongly supported by the validation metrics; the Silhouette score was highest for k = 2 (0.433), and the Davies-Bouldin score was lowest (1.185), both of which confirm that the two-cluster solution provides the best balance of intra-cluster cohesion and inter-cluster separation. Although the Calinski-Harabasz score was highest for k = 3 (4938.07), the powerful and consistent results from the other metrics, combined with the superior clinical interpretability of a binary split, guided our final decision. The two-cluster solution offered the most compelling and clinically actionable dichotomy, a high-complexity, high-resource-utilization group versus a lower-complexity, stable group, which is highly valuable for informing targeted health policies and resource allocation.

For the implementation of patient cluster analysis, the Python programming language was used, with the scikit-learn library (version 1.4.2) as the main tool, which provides robust functionalities for machine learning and data processing. The clustering algorithm chosen was K-Means, due to its computational simplicity, interpretability and efficiency in identifying internal structures in large data sets.

Pre-processing included:

- Selection of relevant variables for segmentation: age, number of comorbidities, total length of hospitalization, frequency of hospitalizations, binary sex and background of origin.
- Scaling these variables using the StandardScaler function in scikit-learn, to ensure comparability between variables expressed in different units.
- The K-Means model has been initialized with the following parameters:
- n_clusters = 2: value determined empirically as optimal by the Elbow and Silhouette methods.
- random_state = 42: for reproducibility.
- n_init = 10: the number of repetitions of the algorithm with different initial centers, to avoid local minimum.
- max_iter = 300: The maximum number of iterations per run.

The training of the model was achieved by calling the fit_predict method [19] on the scaled dataset, resulting in a cluster membership vector that was later added to the original dataset for descriptive profile analysis.

This approach has made it possible to achieve a coherent segmentation of the population, based on clinically significant and easily obtainable traits from administrative sources.

#### 2.4.2. Statistical Considerations for Data Analysis

For the analysis of quantitative data, the dispersion of variables was described by means of the interquartile range (IQR) [22], providing a robust picture of variability in the case of asymmetric distributions. This measure was preferred in the context of the presence of deviations from normality, being complementary to the median values in the representation of the central trend.

The evaluation of the statistical significance of the differences between the groups was carried out by calculating the *p*-value [23], establishing a conventional significance threshold of 0.05. In the case of categorical variables, the Chi-square test [24] was used to investigate associations between groups. For the comparison of distributions, the non-parametric Mann–Whitney U test [25] was applied, suitable for independent samples and continuous data with non-normal distributions.

The normality of the distributions was verified prior to the application of inferential tests, using the Shapiro-Wilk test [26]. This approach allowed the selection of the most appropriate statistical tests, avoiding the incorrect application of parametric methods in the context of data that do not comply with the normality hypothesis.

## 3. Results

### 3.1. General Characteristics of the Population

The total database, for the 6 years of the study, consists of 116,264 hospitalizations due to chronic ulcers, generated by 69,349 patients. Of these, 50,493 patients had only one hospitalization during the study period and were excluded from the analysis. The remaining database consists of 65,771 hospitalizations, generated by 18,856 patients who were hospitalized two or more times for chronic ulcers during this period. Thus, this database is considered valid and is the subject of our study.

The median age of the patients was 68 years, with an amplitude between 18 and 100 years, which indicates a predominant impairment of the elderly population, which is in a biological stage vulnerable to chronic healing processes.

From the perspective of gender distribution, the ratio was 54.5% male patients, with an absolute number of 35,864 men and 29,907 women, which suggests a slight male preponderance among hospitalized cases.

As regards socio-professional status, there was a clear dominance of retired patients (79.5%), followed by salaried and unemployed categories (9.9% each), the rest being marginally represented: self-employed (0.4%), unemployed (0.3%), pupils or students (0.08%), employers (0.04%) and farmers (0.02%). These data reflect a population with a high degree of dependence on the public health system, especially among retirees.

The geographical distribution of patients showed a slight predominance of rural areas (56.9%) compared to urban areas (43.1%). This distribution may be associated with unequal access to specialized medical services and difficulties in the preventive and curative management of chronic injuries in rural areas, where medical resources are often more limited.

These general characteristics indicate that the population affected by chronic ulcers is a vulnerable one, with a significant burden of disease associated with old age, comorbidities and precarious socio-economic status, which justifies the application of differentiated management and resource allocation strategies.

### 3.2. Preliminary Descriptive Analysis

**Figure 2:**
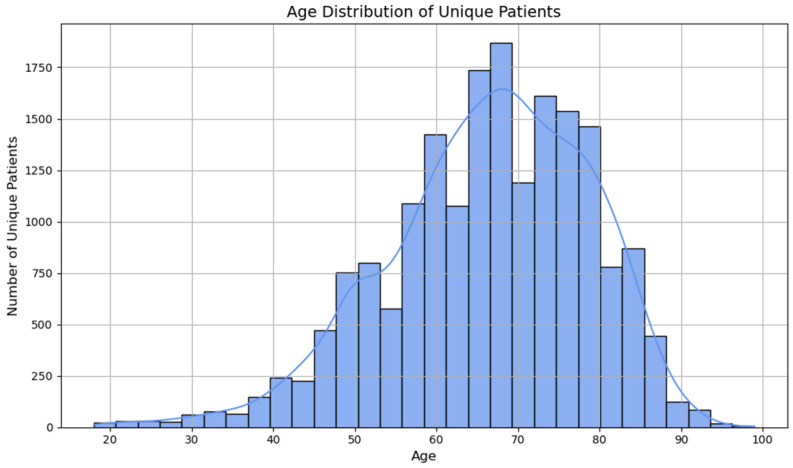

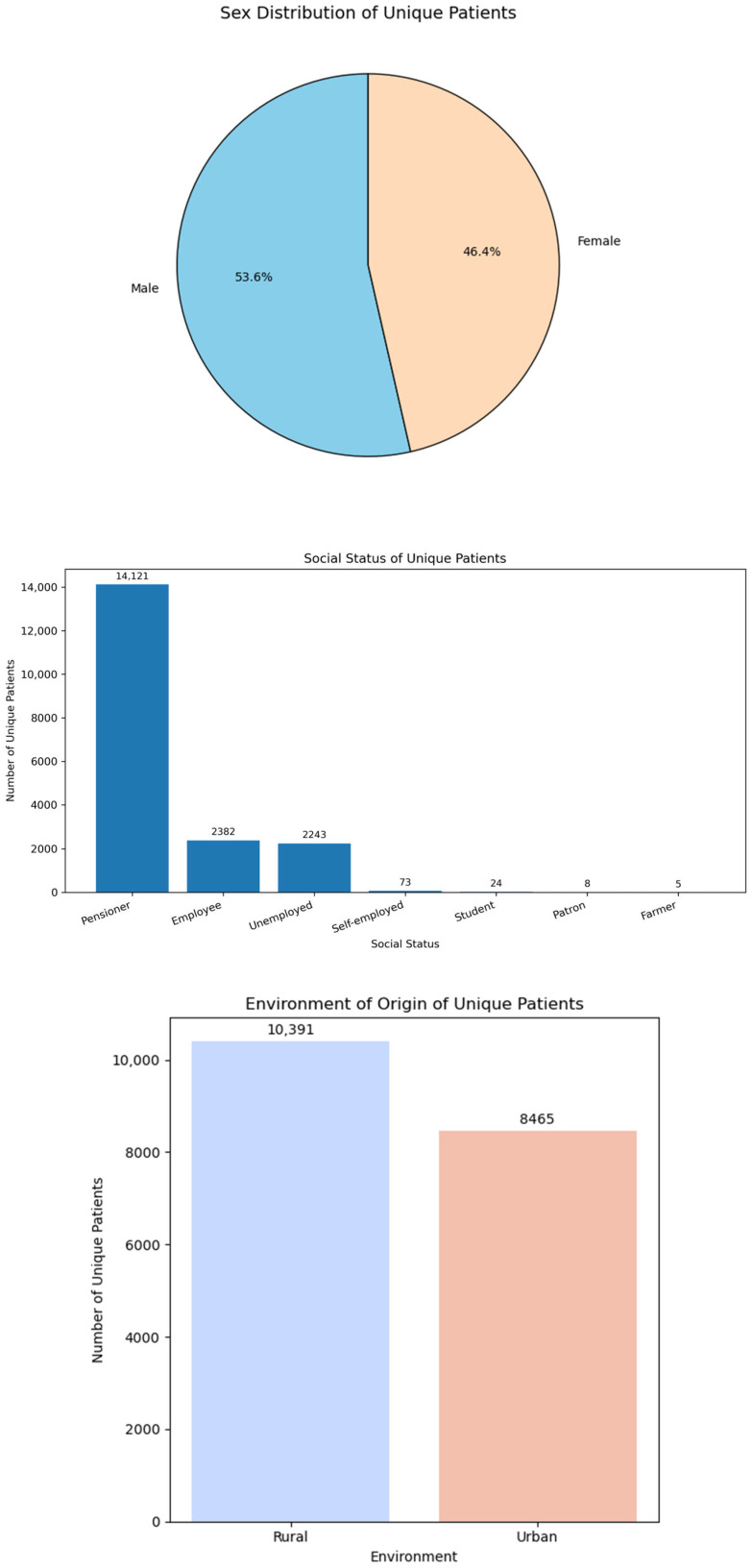
Demographic profile of patients with chronic ulcers.

Descriptive analysis was the first essential step in characterizing the population of unique patients diagnosed with chronic ulcers and hospitalized during the study period. The main demographic, clinical and resource consumption characteristics were assessed, both overall and stratified by ulcer types, sex and age groups. (Figure 2)

In the entire batch of 18,856 unique patients, the median age was 68 years (IQR: 59–76), with a minimum of 18 and a maximum of 100 years. The age distribution is positively asymmetrical (straight tailed), with a peak around the age of 70, reflecting the prevalence of chronic ulcers among elderly patients.

The gender distribution showed a male: female ratio of 53.6%: 46.4%, indicating a slight predominance of male patients. This distribution is constantly maintained by most age groups and is also found in the distributions by clinical categories.

As for the social status of single patients, 74.9% were classified as pensioners, 12.6% as employees, 11.9% without employment, and the rest were distributed among self-employed workers (0.4%), students (0.1%), employers (0.04%) and farmers (0.03%). This structure directly reflects the elderly and vulnerable profile of the population studied.

The distribution according to the environment of origin showed that 55.1% of the patients come from rural areas, and 44.9% from urban areas. This rural predominance may suggest favorable factors such as delayed access to medical care, poor socio-economic conditions and low level of health education, factors that can contribute to the aggravation and chronicity of ulcerative lesions.

It is important to note that the 18,856 unique patients generated a total of 65,771 admissions, which corresponds to an average number of 3.5 admissions per patient, indicating the frequency of relapses and the chronic nature of these conditions.

**Figure 3:**
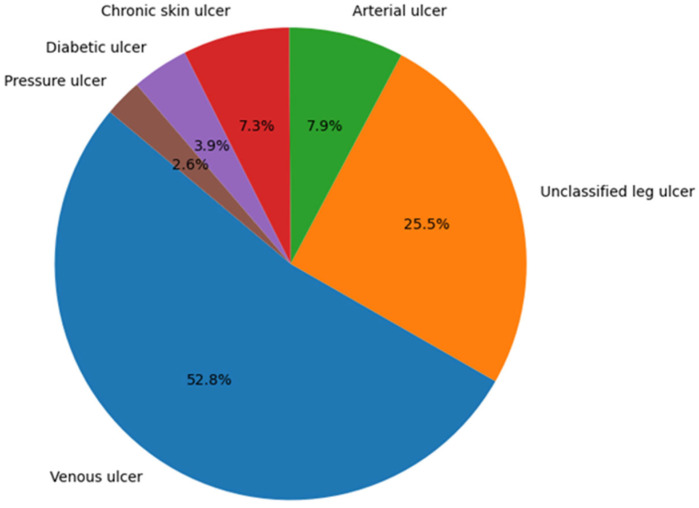
Distribution of hospitalizations by categories of chronic ulcers.

The evaluation of the number of admissions according to the clinical categories of ulcers highlights the fact that the most common types are venous ulcers and lower limb ulcers not elsewhere classified, which together account for over 78% of the total of 65,771 admissions. (Figure 3)

The distribution of admissions is as follows:

Venous ulcer (I83.x): 52.8% of the total (34,751 admissions)
Ulcer of the lower limb not elsewhere classified (L97): 25.5% (16,777 admissions)
Arterial ulcer (I70.23): 7.9% (5173 admissions)
Chronic skin ulcer not elsewhere classified (L98.4): 7.3% (4816 admissions)
Diabetic ulcer (E1x.73): 3.9% (2559 admissions)
Pressure ulcer (L89): 2.6% (1695 admissions)

The number of admissions per patient is shown in Table 2 along with the overall distribution of the number of hospitalization episodes in the analyzed dataset. Patients are assigned to the category after the first episode, and the average number of admissions/patients is calculated for admissions with that main diagnosis. For each patient, the clinical category (type of ulcer) from his first admission was determined. The average number of admissions for a clinical category is calculated only based on admissions in which the main diagnosis is exactly the type of ulcer identified at the patient’s first admission. Any subsequent hospitalization of the patient in which the main diagnosis is different from the initial one is not included in this calculation. Thus, this indicator reflects how frequently the patient was hospitalized strictly for the specific type of ulcer initially identified and does not include any subsequent hospitalizations for other types of ulcers or other conditions.

**Figure 4:**
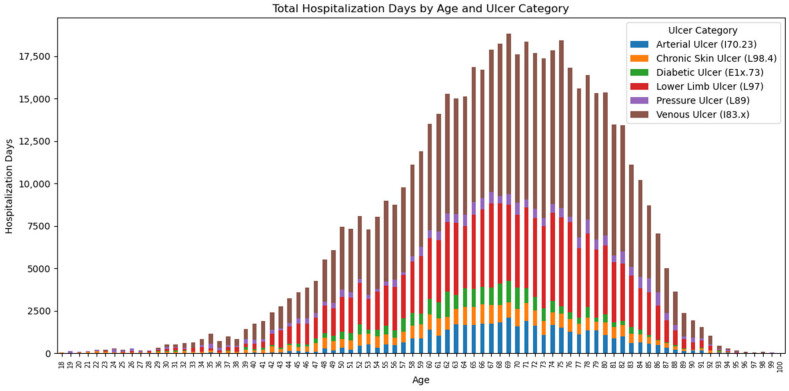
Days of hospitalization by age and category of ulcers.

The total distribution of hospitalization days is illustrated in Figure 4 according to the age of the patients and the clinical category of the ulcer. It is observed that most days of hospitalization are associated with patients aged between 65 and 80 years, indicating a major consumption of resources among the elderly. The categories of ulcers with the greatest impact on the total duration of hospitalization are:

- Venous ulcer, which dominates the entire distribution and reaches a peak between 70–75 years.
- Ulcer of the lower limb not elsewhere classified, with a broad profile around the age of 65–80 years.
- Arterial ulcer and diabetic ulcer, which contribute especially to the age range 60–75 years.
- Bedsores and chronic unclassified skin ulcers have a smaller but constant contribution in the advanced age categories.

**Figure 5:**
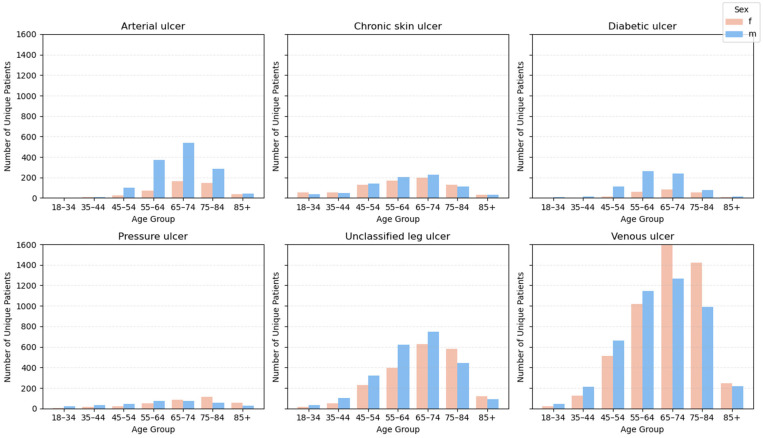
Distribution of patients by categories of ulcers, age groups and sexes.

The distribution of unique patients by age group and sex is detailed in Figure 5 stratified by each clinical category of ulcers. It is noted that:

- Venous ulcer and lower limb ulcer not elsewhere classified (L97) reach peaks in incidence in the age groups 65–74 years and 75–84 years, in line with the advanced mean age of the population studied. Based on the analyzed data, it was found that, of all the clinical categories of chronic ulcers studied, only venous ulcers present a clear predominance of female patients.
- For arterial and diabetic ulcers, a clearly favorable distribution of the male sex is observed, with proportions of approximately 74.2% and 77.8% of the total cases analyzed
- Bedsores and chronic unclassified skin ulcers have a more balanced distribution between the sexes, with a slight male predominance in older age groups.

**Figure 6:**
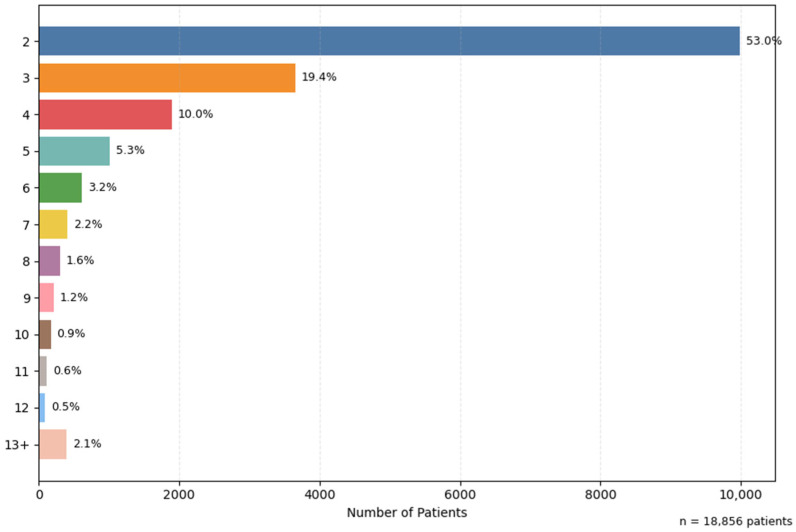
Number of hospitalizations per patient.

The analysis of the number of hospitalizations per patient showed an overall average of 3.5 admissions/patient, indicating a high number of recurrences. About 53% of patients had two admissions, and 12.3% were readmitted more than five times. This suggests that the database does not only reflect isolated hospitalizations, but consistently includes recurrent cases, relevant for assessing the chronic burden on the health system. (Figure 6)

**Figure 7:**
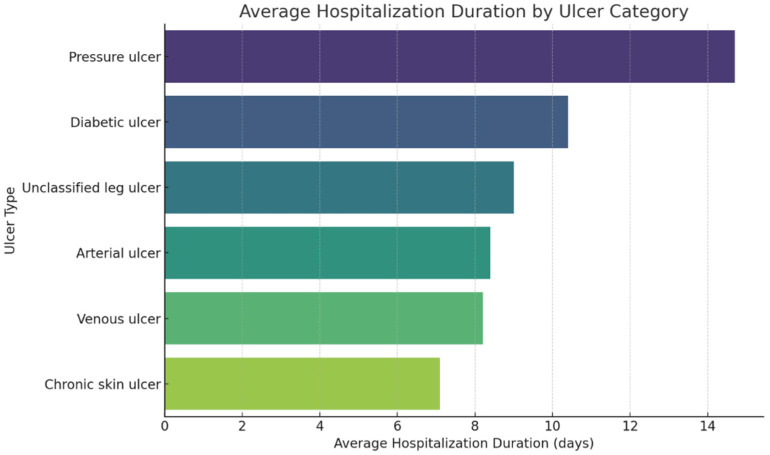
Average number of days of hospitalization for the 6 categories of ulcers.

The median number of days of hospitalization per admission was 8 days (IQR: 6–10), the average length of a hospital stay was 8.6 days. (Figure 7)

The analysis of the distribution of consumed resources highlighted significant differences between the clinical categories of chronic ulcers, mainly reflected by the total duration of hospitalization and the average number of hospitalizations per patient, used as relevant indicators for assessing the burden on the health system.

The average duration of hospitalization per admission demonstrated significant variation based on ulcer type. The longest mean stay was observed for bedsores (L89), at 14.7 days, followed by diabetic ulcers (E1x.73) at 10.4 days. Lower limb ulcers not classified elsewhere (L97) averaged 9.0 days, arterial ulcers (I70.23) 8.4 days, venous ulcers (I83.2) 8.2 days, and chronic skin ulcers not otherwise specified (L98.4) had the shortest average stay at 7.1 days.

This distribution suggests a correlation between the severity and complexity of cases and the duration of hospital management. The differences are further reflected in the age group distribution, with the highest average ages observed among patients with arterial ulcers (69.0 years), followed by venous ulcers (67.6 years), decubitus ulcers (67.0 years), lower limb ulcers not classified elsewhere (67.1 years), and diabetic ulcers (64.7 years). In contrast, chronic unclassified skin ulcers (L98.4) occur at a significantly lower average age of 62.6 years. This pattern may indicate that L98.4 is more common in younger patients with milder or differently managed forms of ulcerations, whereas advanced age appears to be associated with greater severity and complexity of arterial pathology, as seen in cases coded as I70.23.

Also, recurrence (average number of admissions per patient) ranges from 2.6 admissions in patients with pressure ulcers, to 3.8 admissions in those with unclassified ulcers of the lower limb, indicating a frequent return to the hospital and possible deficiencies in outpatient management or lack of community support.

These differences reflect not only the pathophysiological particularities of each type of ulcer, but also the varying needs for multidisciplinary treatment, prolonged monitoring, surgery and functional recovery. Pressure ulcers and diabetic ulcers often involve patients with multiple comorbidities and poor functional status, which translates into an increased consumption of medical and social resources.

### 3.3. K-Means Clustering Results

The application of the K-means algorithm on the processed dataset led to the identification of two distinct clusters of patients, with significantly different clinical profiles and resource consumption. This segmentation allowed a more nuanced understanding of the population studied, highlighting the existence of subgroups with contrasting medical needs and economic impact. The analysis of the characteristics of each cluster was carried out based on standardized variables, revealing relevant patterns in terms of age, comorbidities, duration of hospitalization and frequency of hospitalizations.

The descriptive details of each identified cluster are presented below, together with the associated clinical interpretations, useful in outlining differentiated directions of intervention in medical practice.

It is essential to emphasize that these values reflect indicators calculated at the level of a single patient, i.e., they are the result of the aggregation of all admissions and diagnosis per individual, and not at the level of hospitalization episode.

#### 3.3.1. Cluster 1—Patients with a Complex Profile, Intensive Consumption of Resources

This cluster is characterized by:

- higher average age, 73.1 years.
- significantly longer total hospitalization duration—these patients accumulated a high number of days in hospital (61.4 days/patient).
- average increased number of comorbidities (4.6).
- higher frequency of readmissions (on average 3.3), indicating a profile with complicated medical evolution and recurrent risk.
- increased probability of rural origin (61.4%) and male (55.8%), and the dominant social status, retired.

Medical interpretation: this cluster includes elderly patients, with marked biological frailty and multiple chronic pathologies, requiring long-term care, repeated hospitalizations and interdisciplinary interventions. They represent a category with a high risk of recurrence and complications, an ideal candidate for integrated chronic disease management, community care and post-discharge coordination programs.

#### 3.3.2. Cluster 2—Patients with a Simpler, Clinically Stable Profile

This cluster includes:

- more varied ages, including those under 60 years old, but generally younger than in Cluster 1, with an average age of 63.4 years.
- reduced total length of hospital stay associated with shorter or well-managed episodes (average total length of hospitalization: 20.9 days/patient).
- comorbidities in fewer numbers (average of 2.6).
- low frequency of admissions (2.1).
- more balanced distribution in terms of gender (male 47.5%), environment of origin (urban 51.1%) and dominant social status (employee).

Medical interpretation: these patients have a clinically stable profile, with a less severe form of the disease and a better ability to be managed on an outpatient basis or in community care. The consumption of resources is significantly lower, and interventions can be focused on therapeutic education, prevention and punctual social support.

As practical and clinical management implications, it follows that patient segmentation allows for differentiated allocation of resources, cluster 1 can benefit from multidisciplinary care, access to advanced specialized treatment centers with prolonged hospitalization, and cluster 2 can be oriented towards outpatient monitoring programs, medical education and recurrence prevention.

The clinical and resource utilization differences between the two clusters of patients obtained by the K-Means algorithm is summarized in Table 3. Each row corresponds to an evaluated parameter (age, total duration of hospitalization, number of comorbidities, frequency of hospitalizations, sex and environment of origin, social status), and the columns describe the characteristic features of each cluster. Cluster 1 highlights an older patient profile, with multiple chronic diseases and increased consumption of resources, while Cluster 2 includes younger patients, with less complex pathology and more favorable clinical evolution. This segmentation can support differentiated resource allocation decisions and personalized interventions.

### 3.4. Analysis of Resource Consumption

**Figure 8:**
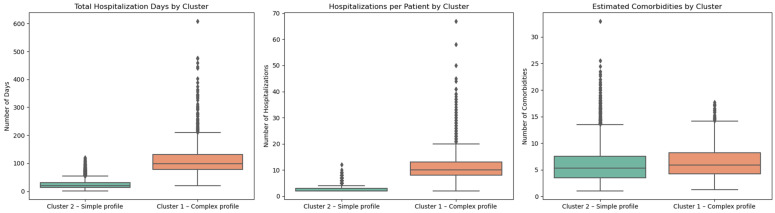
Resource consumption of the two clusters (K-means).

**Figure 9:**
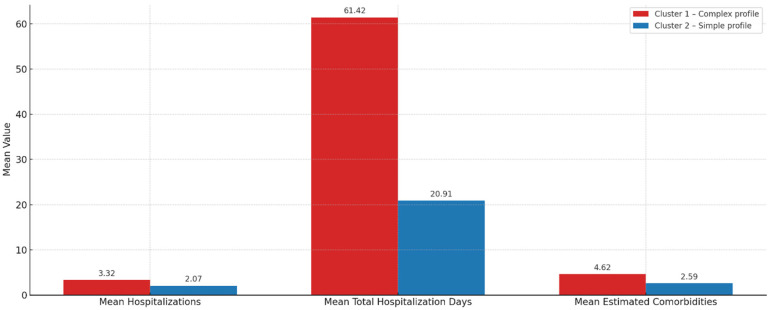
Indicators of the average resource consumption of the two clusters.

The segmentation of the population with chronic ulcers allowed the identification of two distinct groups of patients, significantly differentiated in terms of the consumption of medical resources. Cluster 1 is characterized by increased consumption: patients included in this profile had an average of 3.32 admissions, a mean total length of hospitalization of 61.42 days, and an estimated mean number of 4.62 comorbidities (Figure 8 and Figure 9). Comparatively, patients in Cluster 2 had an average of 2.07 admissions, a total duration of hospitalization of 20.91 days and 2.59 comorbidities.

These differences highlight the high clinical complexity and frailty of patients in Cluster 1, indicating the need for multidisciplinary specialized interventions, continuous monitoring, and integrated long-term care strategies.

In contrast, patients in cluster 2 had on average, a single episode of readmission, a reduced total duration of hospitalization, and a simpler clinical profile with fewer comorbidities. They seem to benefit from a more favorable evolution or efficient outpatient management.

The graphical visualizations (Figure 8 and Figure 9) confirm these differences, highlighting a clear segregation of resource consumption between the two clusters. In the absence of explicit data on costs and detailed medical interventions, these indirect indicators provide a conclusive picture of the health burden generated by each group. Cluster 1 clearly stands out as the most demanding for the health system, justifying priority multidisciplinary interventions and proactive prevention and monitoring strategies.

Figure 8 shows the comparative average consumption of medical resources per patient, calculated for the two clusters identified by the K-means algorithm. The analyzed indicators are the average number of admissions per patient, average total number of hospitalization days per patient, estimated average number of comorbidities.

The graph highlights the effectiveness of the clustering method in highlighting distinct clinical profiles among patients with chronic ulcers. The results can inform decisions about resource allocation, prioritization in medical interventions, and the development of differentiated patient management strategies—such as integration into community care or scheduled admissions for complex cases.

### 3.5. Statistical Significance Tests

For clinical validation of the segmentation of the patient population into distinct clusters, a rigorous analysis of the significance of the observed differences between these groups was required. Appropriate statistical tests were applied to each type of variable, to determine whether the differences identified are medically relevant and do not occur by chance.

For each comparison, the *p*-value was calculated, with the significance threshold set to 0.05. *p*-values below this threshold indicate that the observed differences are statistically significant and cannot be attributed to chance. Thus:

*p* < 0.05 indicates a statistically significant difference;
*p* < 0.01 reflects a strongly significant difference;
*p* ≥ 0.05 suggests the lack of a statistically significant difference;

For the clinical interpretation and validation of the clusters, a comparative statistical analysis was performed. To provide a comprehensive assessment of the differences between groups, our analysis included three key components for each comparison: the p-value, the 95% confidence interval (CI) for the difference, and a measure of the effect size. This multi-faceted approach ensures that our findings are not only statistically significant but also clinically interpretable and relevant. *p*-values were used to determine statistical significance (threshold *p* < 0.05), 95% CIs provide a plausible range for the true difference between groups, and effect sizes quantify the magnitude of these differences. For non-parametric comparisons with the Mann–Whitney U test, the rank biserial correlation was used as the effect size, while for Chi-square tests, Cramér’s V was computed to assess the strength of association.

To evaluate the clinical relevance of the clustering obtained, a comparative statistical analysis was performed between the two groups of patients resulting from the application of the K-means method. The analysis tracked differences in age, gender, background, length of hospitalization, number of comorbidities, and frequency of admissions.

Patients’ age, sex and background: The comparison of age between the two clusters showed a statistically significant difference (*p* < 0.05), which suggests the existence of a predominant group made up of elderly patients, compared to a second younger group. This difference is consistent with the hypothesis that elderly patients have a higher severity of chronic ulcers and an increased likelihood of complications and recurrent hospitalizations. In terms of gender, a significant proportional difference was observed, with cluster 1 having a higher share of male patients (confirmed by the Chi-square test). This observation is in line with the literature that indicates a more frequent association of arterial and diabetic ulcers with the male sex. For the environment of origin, the difference between clusters was also significant, with cluster 1, the most vulnerable, including a higher proportion of patients from rural areas, which could reflect delays in accessing medical care and the lack of outpatient monitoring.

Total duration of hospitalization: The total duration of hospitalization was also significantly higher in one of the clusters (cluster 1) (*p* < 0.01), supporting the hypothesis of a higher consumption of hospital resources. This observation is particularly relevant from the perspective of the efficient management of care resources, as patients in the cluster with prolonged hospitalizations are likely to require complex interventions and long-term treatments.

Number of comorbidities: The analysis showed a significant difference in the number of comorbidities between the two groups, suggesting that one of the clusters consists mainly of patients with multiple pathologies. This information is crucial in individualizing the care plan and understanding the risk of unfavorable evolution or readmission.

Frequency of hospitalizations: Cluster 1, characterized by older patients and with more comorbidities, also had a higher frequency of hospitalizations, which suggests the complexity of the cases, their enduring evolution with the frequent occurrence of complications requiring readmission. The difference between clusters was statistically significant (*p* < 0.05), reinforcing the hypothesis of increased clinical vulnerability in this subgroup.

Analysis of the results of the statistically significant tests confirms the distinct clinical differences between the two identified clusters. The cluster of older patients with multiple associated comorbidities and long hospital stays is a group at high risk of resource consumption and requires targeted integrated management interventions. This statistical stratification provides solid premises for the development of policies for efficient allocation of resources and individualization of care for patients with chronic ulcers.

Continuous numerical variables (age, length of hospitalization, number of comorbidities, frequency of hospitalizations) were analyzed using the Mann–Whitney U test, as their distributions were not normal (Shapiro–Wilk test, *p* < 0.05). The results indicated statistically significant differences between clusters for all these variables (*p* < 0.001), confirming the existence of two distinct subpopulations:

A first cluster, characterized by older patients, with longer hospitalizations, multiple comorbidities and frequent hospitalizations.

A second cluster, consisting of relatively younger patients, with a low consumption of resources and a more stable clinical profile.

The categorical variables (sex, environment of origin) were compared by the Chi-square test, which showed significant differences (*p* = 0.0027 for sex; *p* < 0.0001 for the environment). Thus, a tendency was observed to associate a cluster with a higher proportion of male and rural patients, suggesting a possible relationship between socio-demographic factors and disease severity.

In conclusion, the tests applied confirm the validity of the segmentation performed, demonstrating that the two clusters not only differ statistically significantly, but also that these differences have direct clinical relevance. The results support the use of unsupervised learning profiling as a tool to stratify risk and optimize the management of patients with chronic ulcers.

### 3.6. Sensitivity Analysis Including Single-Admission Cases

To evaluate the impact of excluding single-admission cases on our findings, we performed a parallel clustering analysis on the full dataset of 69,349 patients, including the 50,493 patients who had only one hospitalization. In this expanded cohort, these single-admissions patients had a median length of stay of 7.4 days (IQR: 5–9) and, by definition, a mean of 1.0 admissions per patient. When the K-means analysis was applied to the entire patient population, the optimal solution still converged to two distinct clusters with characteristics comparable to those in the original model: a high-burden cluster (older age, multiple comorbidities, prolonged hospitalizations) and a low-burden cluster (younger, fewer comorbidities, shorter stays). The primary difference observed was a downward shift in the absolute values of resource consumption metrics, driven by the large cohort of single-admission patients. However, cluster structure and separation remained stable, with comparable silhouette scores, indicating that the segmentation approach was robust. These results suggest that our restriction to recurrently hospitalized patients did not distort the relative differentiation of patient profiles but instead refined the analysis to focus on the subpopulation with higher medical complexity and more frequent inpatient needs.

## 4. Discussion

The two-phenotype solution identified in our study, a stable/low-burden group and a complex/high-burden group, echoes findings from the broader chronic wound literature, which consistently reports the existence of patient subgroups with markedly different healthcare needs and outcomes [27]. Notably, the profile of our high-burden cluster, characterized by advanced age and a substantial comorbidity burden, closely mirrors the ‘high-risk’ groups described in studies based on detailed clinical datasets. This correspondence supports the validity of our utilization-based phenotypes as pragmatic proxies for clinical complexity, even when derived from administrative data lacking granular clinical variables. The application of the K-means grouping method allowed the segmentation of the population of hospitalized patients with chronic ulcers into two distinct clusters in terms of demographic, clinical and resource consumption.

Cluster 1 is characterized by a significantly higher median age, an increased number of comorbidities, longer hospitalizations and a higher frequency of readmissions. This profile reflects elderly patients with multiple comorbidities, with advanced clinical forms, with an increased risk of complications and with a low capacity for self-management.

Cluster 2 includes younger patients with fewer comorbidities and a shorter length of hospitalization. These patients seem to be diagnosed earlier or benefit from a higher healing capacity, which suggests a more favorable profile in terms of prognosis and outpatient therapeutic management.

The comparative analysis of the two clusters highlighted a clear correlation between the clinical profile and the consumption of resources, thus the patients in cluster 1 generated a disproportionate consumption of hospitalization days and medical resources, being responsible for most repeated admissions. This group can be considered a significant burden on the healthcare system, requiring long-term interventions, complex treatments, and often reinterventions. In contrast, cluster 2 has a smaller footprint on hospital services, being associated with shorter episodes of care and lower clinical complexity.

This polarization between the two profiles underscores the need for differentiated layering of care, tailored to the patient’s individual risk.

The results obtained are consistent with the data published in the international literature, which highlight the existence of subgroups of patients with chronic ulcers with distinct clinical and risk profiles. Studies [28,29] have shown that elderly patients with high blood pressure, obesity, peripheral vascular disease, and other comorbidities have a higher risk of frequent hospitalization, complications, and increased costs. There are also data [30,31,32,33,34] confirming that a minority of patients generate a significant proportion of the expenses associated with ulcer care, confirming the usefulness of real-world data-based profiling for resource optimization. Despite the clinical importance of patient profiling in chronic skin ulcers, only a few studies on this topic have been conducted or published in Romania to date [35,36,37,38,39].

Segmenting the population based on clinical and demographic characteristics has direct implications for the personalized management of chronic ulcers:

For patients in the high-risk cluster, intensive follow-up protocols, interdisciplinary care (including internists, diabetologists, vascular surgeons, specialist nurses) and early integration into community or palliative care programs should be implemented.

For the low-risk cluster, the focus should be on prevention, medical education, rapid interventions and avoiding the evolution to complicated stages.

This model allows for data-driven clinical triage, which can guide decisions about case routing and resource allocation.

### 4.1. Limitations of the Study

This study has several important limitations: first pertains to the exclusion of patients with a single hospitalization, who represented over half of the initially identified cases. While this strategy was intended to ensure the inclusion of genuinely chronic cases and minimize the risk of incorporating misclassified or miscoded diagnoses, including those affected by DRG-related coding practices, it may have led to an underestimation of the number of milder cases and an artificial inflation of average resource consumption metrics. However, our sensitivity analysis demonstrated that including these single-admission patients did not alter the fundamental structure or interpretability of the two identified patient clusters, although it lowered the overall average burden estimates. These findings support the robustness of our clustering solution while clearly defining the study’s scope as primarily capturing the segment of patients with recurrent and clinically complex disease trajectories.

Second, our administrative data lacks important clinical details such as ulcer size, location, wound duration, and treatment type. This limits our ability to identify detailed clinical sub-phenotypes or directly measure wound severity. While this reduces clinical precision, many relevant comorbidities are captured in the dataset. Therefore, we used the total number of comorbidities per patient as a practical proxy for clinical complexity, in line with the study’s aim to define profiles based on healthcare burden. These burden-based profiles reflect system-level needs and can support early risk assessment and care planning in outpatient settings. However, our data does not provide the details needed to calculate a full Charlson Comorbidity Index [40], which distinguishes between mild and severe forms of disease. Similarly, the profiles are not directly linked to clinical severity scores such as the WIfI classification [41], which may limit the generalizability of our findings to clinically defined phenotypes and their external validity in broader clinical settings.

Third, our dataset lacks hospital or center level identifiers. Because the data were provided as a de-identified national extract, we could not assess or adjust for between-center heterogeneity. This may introduce residual variability in patient profiles due to unmeasured institutional factors. Future research incorporating center metadata or hierarchical modeling could help address this limitation.

Fourth, our dataset is limited to inpatient hospitalizations and does not include data from outpatient visits, community-based services, home care, or pharmacy claims. These care settings represent important components of chronic ulcer management, particularly for ongoing wound care, follow-up, and preventive interventions. The absence of this information restricts our ability to capture the full continuum of care and may lead to an overestimation of the relative burden of inpatient episodes. As such, our findings primarily reflect the healthcare resource use associated with acute exacerbations and complex inpatient needs, rather than the total longitudinal cost or care pathway. This limitation also hinders the derivation of accurate per-patient cost estimates and prevents a comprehensive analysis of cost drivers, service fragmentation, and access disparities across the healthcare system.

Another limitation concerns data governance and transparency. The administrative database is not publicly accessible, and legal restrictions related to patient confidentiality prevent the sharing of raw data. However, to support reproducibility, the statistical code used for clustering and comparative analyses is available from the corresponding author upon reasonable request, subject to institutional approval.

Another important limitation relates to the absence of key clinical care process indicators that may reflect disease severity or complexity, such as ICU/intermediate care exposure, surgical intervention, and referral origin (e.g., primary care, outpatient). These variables are known to influence healthcare utilization and could help distinguish between different clinical pathways within the high-burden group. Although ER admission status was available, we chose not to include it to avoid introducing potential bias from care process decisions that are themselves influenced by patient burden. The absence of these variables may limit the level of detail or refinement achievable in the identified phenotype and limit our ability to identify distinct clinical subtypes (e.g., urgent surgical vs. chronic medical management). Consequently, the identified clusters should be interpreted primarily as utilization-based profiles, reflecting system-level burden rather than clinical complexity per se. Future studies enriched with structured metadata such as ICU stays, procedure codes, and referral origin will be needed to refine these profiles and assess their clinical interpretability.

Furthermore, the selection of a two-cluster solution, while quantitatively supported and clinically actionable, represents a high-level abstraction of the patient population. This approach may mask nuance in ulcer etiology and obscure smaller, distinct sub-phenotypes within the larger ‘complex’ and ‘simple’ profiles. Future studies could explore solutions with a higher number of clusters to investigate this heterogeneity.

As detailed in the Methods section (Section 2), several clinically relevant process and severity-related variables were either unavailable or unstructured in the dataset (e.g., referral origin, procedure/operating-theatre codes, ICU or intermediate-care indicators). Although admission type (emergency vs. non-emergency) was present, it was deliberately excluded from the clustering process to avoid process-of-care leakage. The absence of these variables may reduce the clusters’ ability to reflect differences in clinical severity and could blend distinct care trajectories (e.g., urgent surgical vs. chronic medical cases) within the high-burden phenotype. Accordingly, the resulting clusters should be interpreted primarily as utilization-based rather than clinically defined phenotypes.

When comparing outcomes across clusters, unmeasured heterogeneity, such as ulcer stage or severity, functional capacity, social support, or access to community-based care, may contribute to observed differences. These factors represent residual confounding, and therefore, we refrain from making causal interpretations.

Regarding validation, external validation across hospitals or regions was not possible due to the absence of site level identifiers in the deidentified dataset. Temporal validation was also not performed. While the large sample size may help reduce the influence of extreme cases, no formal sensitivity analysis was conducted to assess the effect of high-utilization outliers. Given the sensitivity of K-means clustering to such extremes, future research should evaluate robustness by excluding the top 1% of cases based on resource use and applying bootstrap-based or temporal validation procedures.

### 4.2. Future Research Directions

To consolidate and extend the applicability of our findings, future research should pursue several complementary directions:

First, prospective studies are essential to address the inherent limitations of retrospective administrative data. Such designs would allow for the direct collection of key clinical wound variables currently unavailable in our dataset, such as ulcer size, anatomical location, chronicity prior to admission, and treatment modality. Including these elements would enhance clinical profiling by enabling the identification of detailed sub-phenotypes and allow the use of structured clinical assessment tools, thereby improving risk stratification, outcome prediction, and external validity. Furthermore, prospective data collection would minimize the risk of misclassification and reduce potential distortions introduced by DRG-driven coding practices. In these prospective studies, any rare instances of missing data would be addressed using robust statistical methods, such as multiple imputation, to limit bias and maintain the integrity of the dataset.

Second, administrative datasets should be enriched by linking them with clinical information from electronic health records, wound registries, and structured clinical assessment tools. Expanding the dataset also to include outpatient care, primary care, home health services, and pharmacy claims would enable a more comprehensive mapping of the patient journey. This broader view would improve cost attribution across care settings, reveal service fragmentation, and provide insights into access disparities.

Third, future studies should investigate more refined clustering techniques to uncover additional subgroups beyond the high- and low-burden clusters identified in our current model. Exploring solutions with more than two clusters may offer improved population segmentation and better reflect the heterogeneity of chronic ulcer patients, including variation in ulcer etiology, progression, and care needs. Methods such as hierarchical clustering, Gaussian mixture models, or density-based algorithms could reveal latent phenotypes that are not visible through simpler approaches, offering greater resolution for tailoring interventions and optimizing care delivery.

Fourth, future work should include formal validation procedures to assess the stability and reproducibility of clustering results. Approaches such as temporal train-test splits and bootstrap resampling would help confirm the robustness of patient profiles across time and subgroups, thereby strengthening the reliability and generalizability of the findings.

Furthermore, our analysis did not include a sensitivity check on the specific impact of high-cost or high-utilization outliers. While the K-means algorithm is generally robust, particularly with large sample sizes, extreme cases could potentially influence the final position of the cluster centroids. Future work could assess the stability of the clusters after excluding, for example, the top 1% of patients based on resource use, to confirm that the core structure of the phenotypes is not driven solely by these extreme cases.

Finally, a key priority is the development of predictive models to forecast outcomes such as complications, or high resource utilization. These models, built using supervised machine learning algorithms, could integrate longitudinal administrative data, including demographic variables, comorbidity profiles, and prior service use. If validated and embedded into clinical workflows, they could support early identification of high-risk patients and enable timely interventions along the patient journey such as early specialist referral and intensified outpatient care. Such strategies have the potential to improve clinical outcomes while reducing avoidable costs.

Together, these research directions aim to deepen understanding of chronic ulcer patient profiles, support the development of personalized and proactive care pathways, and inform smarter planning and resource allocation, ultimately yielding tangible benefits for both patients and health system.

Our study contributes to the growing body of literature on the application of machine learning in chronic wound care. While our approach uses unsupervised clustering on administrative data to identify patient phenotypes at a systemic level, other research focuses on developing AI-driven tools for point-of-care clinical decision support, such as automated diagnostic systems based on computer vision [42]. Integrating these complementary approaches, systemic phenotyping with bedside diagnostics—represents a promising frontier for building a more holistic, data-driven wound care ecosystem.

### 4.3. Clinical and Administrative Implications

Health Policy and Administrative Planning—The segmentation into complex/high-burden and stable/low-burden phenotypes provides a data-driven foundation for health policy and administrative planning. Recognizing that the high-burden group accounts for a disproportionate share of inpatient bed-days and readmissions allows for targeted allocation of resources and capacity. Multidisciplinary wound-care teams, early referral systems, and structured community-based follow-up can be strategically directed toward this subgroup to improve care efficiency and reduce unnecessary utilization.

Clinical Pathways and Case Management—Clinically, these profiles enable early identification of high-risk patients, those presenting with older age, multiple comorbidities, or frequent prior hospitalizations, at the point of entry into the healthcare system. This facilitates enhanced discharge planning, timely specialist involvement (e.g., wound care, geriatrics), and coordinated outpatient or home-based support. Implementing such strategies may prevent complications and reduce avoidable readmissions. However, any operational tools derived from these profiles should undergo prospective validation before being applied in clinical settings.

Monitoring and Quality Improvement—These phenotypes also provide a valuable framework for program evaluation and service monitoring. By stratifying key performance indicators, such as length of stay, readmission rates, and healthcare utilization, according to phenotype, institutions can track progress over time and detect shifts in case mix or service needs, supporting continuous quality improvement.

Limitations and Future Integration—Importantly, the derived phenotypes are not designed as bedside prognostic tools and do not dictate individual treatment decisions. Their clinical deployment would require the development of a separate, prospectively validated classifier capable of mapping early-encounter data to phenotype membership. Such a tool could be integrated into clinical decision-support systems to deliver more personalized, anticipatory care, bridging the gap between population-level insights and individual patient management.

### 4.4. Fairness and Generalisability Considerations

Although this study identifies robust, high-level phenotypes based on healthcare utilization, these clusters may mask important variation across demographic subgroups (e.g., age, sex, or residential background). This raises fairness concerns: if the high burden phenotype disproportionately includes vulnerable populations, administrative or clinical interventions based on these profiles must be carefully designed to avoid reinforcing existing inequities. Furthermore, as hospital-level identifiers were unavailable in our dataset, we could not assess generalizability across different regions or facility types. Future research should explicitly examine subgroup effects and test the stability of these phenotypes across diverse settings before considering practical implementation.

## 5. Conclusions

This study highlights the complexity and practical value of applying modern analytical methods to evaluate the population of patients with chronic skin ulcers. Using the K-Means algorithm, we identified two distinct patient profiles, differing in clinical and socio-demographic characteristics as well as healthcare resource utilization. One profile is characterized by a higher burden on the healthcare system, with advanced age, multiple comorbidities, prolonged hospital stays, and frequent readmissions. In contrast, the second profile comprises younger patients with more favorable clinical outcomes and lower resource consumption.

More broadly, patient profiling represents an essential component of contemporary medicine, aimed at identifying and characterizing subgroups of patients with shared clinical, demographic, behavioral, or genetic traits. By applying advanced statistical techniques such as clustering, multivariate analyses, or machine learning, hidden patterns that are not apparent through conventional descriptive analysis can be uncovered. In the context of chronic ulcers, this approach reveals that although patients share a common diagnosis, subgroups defined by diabetes, venous insufficiency, or immobility exhibit distinct risk profiles and therapeutic needs, which necessitate tailored management plans. Profiling supports treatment personalization, aligns interventions with subgroup-specific needs, and increases therapeutic effectiveness. Additionally, by identifying the most vulnerable patients, those at higher risk of complications, recurrence, or heavy resource use, it enables more efficient allocation of resources and the implementation of preventive measures that reduce costs and enhance care quality.

A key strength of this study lies in its patient-level analysis, aggregating all admissions and diagnoses per individual rather than treating each hospitalization as a separate episode. This methodology provides a more accurate assessment of the chronicity and complexity of each patient’s condition and avoids overestimating case numbers due to repeated admissions of the same individual. Furthermore, the study delivers a detailed profile of patients with chronic ulcers, highlighting differences in age and ulcer types that can inform personalized care strategies and resource planning. The use of a comprehensive database enhancing several years and diverse ulcer etiologies further enhances the robustness and relevance of the findings, contributing to a more nuanced understanding of this heterogeneous patient population.

This segmentation has direct implications for public health policy and hospital management, offering a deeper understanding of how complex cases are distributed within the healthcare system and providing a solid foundation for targeted, differentiated interventions. Patient profiling enables the early identification of individuals who require intensified monitoring, timely interventions, and prioritized resource allocation. Such an approach promotes more efficient use of hospital capacity and budgets, ultimately improving both system performance and patient outcomes.

In summary, patient profiling is an indispensable tool for advancing personalized medicine, optimizing healthcare resources, and enhancing the efficiency and sustainability of health services. Its benefits are evident both at the patient level, through improved clinical outcomes and quality of life, and at the system level, through cost reduction and better strategic planning of interventions.

## Figures and Tables

**Table 1:** Validation metrics.

Number of Clusters (k)	Silhouette Score (Higher Is Better)	Davies-Bouldin Index (Lower Is Better)	Calinski-Harabasz Index (Higher Is Better)
2	0.433	1.185	4277.60
3	0.243	1.509	4938.07
4	0.245	1.318	4744.81
5	0.305	1.309	4756.70

**Table 2:** Descriptive situation of the dataset (I83.x—Venous ulcer; L97—Ulcer of the lower limb not elsewhere classified; I70.23—Arterial ulcer; L98.4—Chronic skin ulcer not classified elsewhere; E1x.73—Diabetic ulcer; L89—Decubitus ulcer; n—number; H—hospitalizations; P–patients).

Ulcer Type	n. H	% of Total	Average Age	n. P	% of Men	Average n. H/P	Average Days/H
I83.x	34,751	52.8	67.6	9503	47.8	3.66	8.2
L97	16,777	25.5	67.1	4373	53.9	3.84	9
I70.23	5173	7.9	69	1804	74.8	2.87	8.4
L98.4	4816	7.3	62.6	1562	51.2	3.08	7.1
E1x.73	2559	3.9	64.7	952	76.9	2.69	10.4
L89	1695	2.6	67	662	49.1	2.56	14.7

**Table 3:** Interpretation of the comparative table between clusters.

Evaluated Parameter	Cluster 1	Cluster 2
Age	significantly higher age (73.1 years), suggesting a predominance of elderly patients likely affected by advanced chronic diseases and long-standing, slow-healing ulcers with multiple complications	lower age (63.4 years), includes younger patients with which may reflect earlier forms of ulcers or better healing and compensation capacity
Sex	the average of binary values indicates a male preponderance—55.8%	a better or even slightly feminine balance—52.5%
Total days of hospitalization	significantly higher total days of hospitalization (61.4 days/patient), indicating the need for more complex care and increased consumption of resources	lower total day of hospitalization (20.9 days/patient), indicating shorter episodes and fewer admissions
Comorbidities number	high number of comorbidities (4.6), which increases the complexity of the cases	fewer associated diseases (2.6) that are likely to be easier to manage
Hospitalizations frequency	high number of hospitalizations (3.3 times) due to difficulties in healing and the need for ongoing monitoring or treatment of complications	lower number of hospitalizations (2.1 admissions), supporting the idea of a clinically stable profile
Environment of origin	higher proportion of patients from rural areas (61.4%), where access to prevention and early health services is often limited	has more patients from urban areas (51.1%), who can access medical care and continuous monitoring more quickly
Social status	it is categorically dominated by retired patients, the percentage of unemployed patients is low, and the proportions of employees and other social categories are extremely low, confirming the limited and vulnerable socioeconomic profile	have a more diversified socioeconomic distribution, with most patients being retired, but with a significant proportion of employees and unemployed people

## Data Availability

Dataset available on request from the authors. The raw data supporting the conclusions of this article will be made available by the authors on request.
